# Nasogastric Feeding Tube/Dobhoff Placement: A Multidisciplinary Approach to the Management of Malnutrition During Radiation Therapy in Patients With Head and Neck Cancer

**DOI:** 10.7759/cureus.24905

**Published:** 2022-05-11

**Authors:** Alex Coke, Marissa Gilbert, Sue Hill, Farzan Siddiqui

**Affiliations:** 1 Radiation Oncology, Henry Ford Health System, Detroit, USA; 2 Nutrition, Henry Ford Health System, Detroit, USA

**Keywords:** dobhoff, nasogastric tube, enteral nutrition, radiation therapy, malnutrition, head and neck cancer

## Abstract

Background

Radiation therapy (RT)-associated oral mucositis, xerostomia, thick mucoid saliva, nausea/vomiting, and loss of taste may result in significantly compromised oral intake in patients undergoing treatment for head and neck cancers (HNC). Feeding tube placement allows patients to receive enteral nutrition and continue the planned course of treatment.

Objectives

RT-associated oral mucositis, xerostomia, and loss of taste may result in significantly compromised oral intake in patients undergoing treatment for head and neck cancers. We sought to determine if reactive nasogastric (NG) tube placement was an effective strategy for nutritional support in these patients and if invasive percutaneous endoscopic gastrostomy (PEG) tube insertion could be avoided.

Methods

This is an institutional review board (IRB)-approved study of patients treated for head and neck cancer using definitive or adjuvant RT with or without concurrent chemotherapy between June 2017 and December 2020. We evaluated the indications for NG tube (Dobhoff) placement, time of placement during the course of RT, patient tolerance of NG tube, and median duration of NG tube placement. In addition, we followed weight loss during treatment, treatment interruptions, and treatment-related toxicities. Complications associated with having the NG tube, if the NG tube needed to be replaced during treatment, and if the patient had any hospitalization during the course of treatment were tracked.

Results

Of the 441 patients treated for head and neck cancer during the time period of this study, 47 required reactive NG tube placement for nutritional support. Patients included 40 with primary oropharynx, three with oral cavity, two with larynx, one with nasopharyngeal, and one was unknown. Chemotherapy was given concurrently with radiation in 87.2% (n=41) patients. The median time of NG tube placement was during Week 5 of the six to seven-week course of RT. The median percentage of weight loss from baseline to the date of NG tube placement was 12.9% (range, -0.9% to 25.9%). The median rate of weight loss decreased by 8.7% from the date of NG tube placement to the end of treatment. The median duration of NG tube placement was 29 days (range, 5 to 151 days). There were no serious medical complications associated with having the NG tube in place during treatment. Twenty-seven point six percent (27.6%; n=13) of patients had the NG tube dislodged or displaced and needed replacement. Thirty-eight point three percent (38.3%; n=18) of patients with an NG tube had to be hospitalized during the course of RT, with the predominant symptoms being failure to thrive (22.2%; n=4) and nausea/vomiting 22.2% (n=4). Six point four percent (6.4%; n=3) of patients requested the removal of the NG tube due to local irritation. Seventy-six point six percent (76.6%; n=36) of patients did not require further nutritional support with the placement of a percutaneous endoscopic gastrostomy (PEG) tube.

Conclusion

This study indicates that clinic placement of an NG tube for patients receiving RT for head and neck cancer is a safe and effective way to maintain nutrition during treatment. The rate of weight loss decreased after the patient had an NG tube placed. The placement procedure is well-tolerated and there were no complications associated with having the NG tube during treatment. PEG tube insertion was avoided in approximately 80% of the patients.

## Introduction

In 2021, an estimated 68,630 new cases of head and neck cancer (HNC) occurred in the United States, with 14,620 deaths resulting from this cancer [1]. The majority of patients with HNC had radiation therapy (RT) as part of their treatment. RT can be given alone or in combination with chemotherapy. In most cases, if surgery is needed, RT is given four-six weeks after surgery. For HNC treatment, the total RT duration is between six and seven weeks. One of the main goals during treatment is to minimize the long-term consequences of side effects experienced during treatment.

A majority of the side effects (SEs) from radiation affect the aerodigestive system. The most commonly experienced SEs include mucositis/mouth sores, dry mouth, thickening of secretions, nausea/vomiting, dysphagia, odynophagia, and loss of taste. These SEs usually begin between weeks two and three of treatment and can contribute to decreased oral intake and increased risk for weight loss and malnutrition. To help decrease the severity of these expected SEs, pain medications are used during treatment to improve pain levels and aim to improve the patient's ability to eat and drink. Mouth rinses can also be used during treatment to help clear oral secretions before eating. These toxicities can be acute and last several weeks post-treatment while additionally contributing to decreased PO (per os) intake and malnutrition. These toxicities can lead to treatment breaks that can impact disease control. The tumor control rate is 1.4% lower for every day that RT is interrupted [2]. In addition, there is a higher likelihood of local recurrence when treatments are missed [3].

One of the most effective ways to maintain adequate nutrition in patients undergoing radiation is the use of enteral tube feeding (ETF). The most commonly used method to deliver ETFs is the use of a percutaneous endoscopic gastrostomy (PEG) tube or nasogastric (NG) tube. We sought to determine if reactive NG tube placement in the clinic was a feasible and effective strategy for nutritional support in these patients. Our study looked at the current process of placing NG tubes, specifically Dobhoff tubes, in the Radiation Oncology clinic and how this affected patient experience and outcomes.

## Materials and methods

This is an institutional review board (IRB)-approved, single-institution, retrospective study. Approval was obtained through Henry Ford Health Systems’ IRB committee under IRB#8751, which includes a full waiver of consent. The patients were first identified using a departmental dataset maintained by an HNC dietitian (RD). This dataset contains patients diagnosed with an HNC between June 2017 and December 2020 who received a Dobhoff. Patients were treated using definitive or adjuvant RT with or without concurrent chemotherapy. These patients were then queried from a separate institutional HNC database (REDCap 10.6; Nashville, TN) generated from the weekly multidisciplinary tumor board for information regarding demographics, diagnosis, treatment, weight, feeding tube placement and duration, hospitalizations, and follow-up.

We evaluated the indications for Dobhoff placement, time of placement during the course of RT, patient tolerance of Dobhoff, and median duration of Dobhoff placement. In addition, we evaluated weight loss during treatment, treatment interruptions, and treatment-related toxicities. Complications associated with having the Dobhoff, if the Dobhoff needed to be replaced during treatment, and if the patient had any hospitalization during the course of treatment were documented.

The indication for Dobhoff placement is significant weight loss, losing >= 10% of pre-treatment body weight, during the course of treatment. Most often, this weight loss is due to dysphagia and/or odynophagia unrelieved by pain medication. Dobhoff placement is done in the radiation oncology outpatient clinic by the attending physician and/or nurse practitioner (APRN). The total procedure time is about 10 minutes as described below. This process is described in Table 1. Patients are able to use the feeding tube immediately once the supplies are delivered (within 24-48 hours).

**Table 1:** Instructions for Nasogastric Feeding Tube Placement

Step	Instructions
1	Verbal consent for the procedure was obtained. Place the patient in a clinic procedure chair with the head in a flexed position.
2	Deliver two puffs of Afrin and two puffs of 4% lidocaine to both nostrils.
3	Select a small bore (10 French) silicone nasogastric feeding tube (Dobhoff) with an oral tip syringe.
4	Take a measurement from the tip of the nose, around the ear, and down halfway between the xiphoid process and umbilicus. Use this measurement to determine the length of the tube needed to be advanced in order for correct placement.
5	Lubricate the tip of the tube.
6	Advance the tube through the right or left nare to the goal length previously measured in step 4. Encourage deep breathing techniques and small sips of water taken through a straw to assist the tube in correct placement.
7	Before placement has been confirmed, a temporary tape is placed on the top of the nose at the half split length and wrapped around the tube. An additional piece is placed across the bridge of the nose.
8	Confirm the correct placement of the tip of the feeding tube in the stomach with an X-ray of the kidney, ureter, and bladder.
9	After placement has been confirmed, a permanent dressing is secured. The skin on the bridge of the nose is prepped with non-sting adhesive. A nasogastric tube holder adhesive is secured to the top of the nose pad. The two long pieces are wrapped around the Dobhoff for additional securement. A multi-purpose tube holder is placed on the patient's chest to further secure the lower portion of the tube.
10	Feeding tube care and use are demonstrated by the dietician to the patient and caretaker.

After advancing the tube into the hypopharynx, direct visualization with a nasopharyngoscope was performed by an additional trained provider (attending physician). This is done simultaneously to confirm the tube is not placed in the trachea. The use of direct visualization is an optional step in the placement procedure, which gives the ability to visualize the internal anatomy and the ability to change the tube direction as indicated/needed. NG tubes placed using direct visualization show an overall placement time in the five to 11-minute range with 90% gastric placement [4]. Radiographs to confirm Dobhoff placement are currently the gold standard in practice [4]. An optimal Dobhoff securement technique is imperative for successful continued use and the prevention of dislodgement/interruption of nutrition.

As described, a multidisciplinary approach is integral to the successful and timely placement of Dobhoff tubes. A variety of clinicians are able to perform Dobhoff placement depending on the institutional guidelines and certain licensure requirements. This may include nurses, advanced practice registered nurses (APRN), dieticians (RD), physician assistants, resident physicians, and primary/attending physicians. At our institution, placement is primarily performed by the APRN with the assistance of the primary attending physician.

## Results

Disease characteristics 

Of the 441 consecutive patients treated during the time period of this study, 47 required reactive Dobhoff placement for nutritional support. Patient demographics and disease characteristics for this cohort can be found in Table 2. The median age of diagnosis for an HNC was 61 years old (range, 43-71 years) and patients were predominantly male (n=39, 83.0%). Primary tumor sites included oropharynx (n=40, 85.1%), oral cavity (n=3, 6.38%), larynx (n=2, 4.26%), nasopharyngeal (n=1, 2.13%), and unknown (n=1, 2.13%). Most patients presented with locally advanced disease (n=28, 60.0%). Human papillomavirus (HPV) status was positive in 78.7% (n=37) of patients, negative in 10.6% (n=5) of patients, and unknown or not tested in 10.6% (n=5) of patients. Chemotherapy was given concurrently with radiation in 87.2% (n=41) patients, where the majority of these patients received definitive chemoradiation (n=35, 74.5%).

**Table 2:** Patient Characteristics HPV, Human Papillomavirus

	N = 47	%
Age at Diagnosis		
<50 years	5	10.6
>= 50 and <60 years	15	31.9
>=60 and <70 years	19	40.4
>=70 years	8	17.0
Gender		
Male	39	83.0
Female	8	17.0
Primary Tumor Site		
Oropharynx	40	85.1
Oral Cavity	3	6.38
Larynx	2	4.26
Nasopharyngeal	1	2.13
Unknown	1	2.13
Stage		
Localized	19	40.0
Locally Advanced	28	60.0
Treatment Modality		
Definitive Radiation	4	8.51
Definitive Chemoradiation	35	74.5
Postoperative Radiation	2	4.26
Postoperative Chemoradiation	6	12.8
HPV Status		
Positive	37	78.7
Negative	5	10.6
Unknown	5	10.6

Feeding tube characteristics 

Data pertaining to feeding tube characteristics can be found in Table 3. The median duration of Dobhoff placement was 29 days (range, 5-151 days). The majority of patients (n=29, 61.7%) required Dobhoff placement between the third and sixth week of their RT course, with the median time of placement occurring in the fifth week of treatment. The median percentage of weight loss from baseline to the date of Dobhoff placement was 12.9%, with most patients experiencing a weight loss between 20 and 30 pounds.

**Table 3:** Feeding Tube Characteristics NG, Nasogastric; PEG, Percutaneous Endoscopic Gastrostomy

	N = 47	%
Week of NG Tube Placement		
<3rd week	7	14.9
>=3rd and <6th week	29	61.7
>=6th week	11	23.4
Percent Weight Loss from Start of Treatment to NG Tube Placement		
Gained	1	2.1
<5%	7	14.9
>=5% and <10%	8	17.0
>=10% and <15%	15	31.9
>=15% and <20%	9	19.2
>=20%	7	14.9
Percent Weight Loss from NG Tube Placement to End of Treatment		
Gained	4	8.5
<5%	24	51.1
>=5% and <10%	14	29.8
>=10% and <15%	3	6.4
>=15% and <20%	1	2.1
>=20%	1	2.1
Days with NG Tube		
<15	14	29.8.
>=15 and <30	10	21.3
>=30 and <45	15	31.9
>=45 and <60	2	4.26
>=60 and <75	2	4.26
>=75	4	8.51
PEG Required After Dobhoff		
Yes	11	23.4
No	36	76.6

Following Dobhoff placement, the median percentage of weight loss from the date of insertion to the completion of treatment was 4.2%, with most patients experiencing a weight loss of less than 10 pounds. Eleven patients (23.4%) required additional nutritional support which resulted in the placement of a PEG tube. Indications for additional nutritional support include patient preference and median percent weight loss greater than 10%.

Feeding tube complications

There were no serious medical complications associated with having the Dobhoff in place during or after treatment. Feeding tube-associated complications and hospitalizations during the course of treatment are summarized in Table 4. Fifteen patients (31.9%) had the Dobhoff dislodged, displaced, or clogged. Eight of these patients (17.0%) required Dobhoff replacement. Three patients requested the removal of the Dobhoff due to local irritation, with one of these patients further requiring a PEG tube. Eleven patients with a Dobhoff required hospitalization for uncontrolled nausea and vomiting (n=3), diarrhea (n=3), and other cardiac and pulmonary causes during the course of RT.

**Table 4:** Feeding Tube Complications PEG, Percutaneous Endoscopic Gastrostomy

	N = 47	%
Successful Placement of Dobhoff		
Yes - No Further Action	46	97.9
No - Required Advancement	1	2.13
Dobhoff Malfunctions		
Dislodged/Displaced	13	27.7
Clogged	2	4.26
Requested Removal	3	6.38
None	29	61.7
Hospitalizations		
Yes - PEG	7	14.9
Yes - Dobhoff	11	23.4
No	29	61.7

Follow-up 

**Figure 1:**
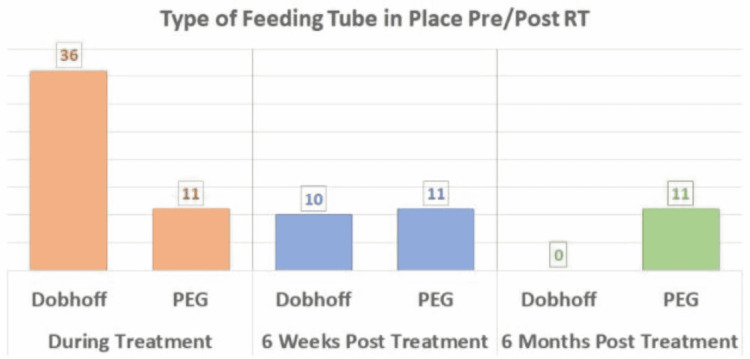
Feeding Tube Placement Pre/Post Treatment RT, Radiation Therapy; PEG, Percutaneous Endoscopic Gastrostomy

At the six-week follow-up appointment, 26 patients (55.3%) were without a feeding tube, 10 patients (21.3%) still required a Dobhoff, and all 11 PEG patients (23.4%) still required their PEG tube. At the six-month follow-up appointment, the majority of patients were without a feeding tube (n=36, 76.6%). No patients had a Dobhoff (n=0) while all 11 PEG patients still required their PEG tube (23.4%). This information is visualized in Figure 1.

All data are available on reasonable request.

## Discussion

Prophylactic PEG vs. reactive PEG

There is currently no standard of care regarding the timing of feeding tube placement for a patient undergoing radiation for HNC. Many studies agree PEG tube placement should be selective or reactive, not routine. Some studies have looked to compare outcomes when patients have a feeding tube placed before symptoms begin (prophylactic placement) versus after symptoms have occurred (reactive placement). A systematic review aimed to compare outcomes in HNC patients with prophylactic PEG (pPEG) versus reactive PEG (rPEG) placement while receiving chemoradiotherapy (CRT) [3]. In some studies, average weight loss at different time points during treatment seemed to be similar in both scenarios.

On the contrary, certain groups of patients studied were found to have higher weight loss and malnutrition with rPEG. The reviewed studies found no difference in disease control and no difference in treatment delays between pPEG and rPEG. One such study found patients receiving rPEG tubes had them in place for fewer days than pPEG [5]. One study found that patients who received rPEG had a higher two-year rate of aspiration and stricture rate. The literature showed patients who received a pPEG tube had a statistically significant improvement in quality of life at six months post-treatment but a decreased quality of life during and at the completion of treatment. Additionally, a correlation was found that resulted in fewer hospitalizations from nutritional deficits with the use of pPEG tubes in comparison to rPEG tube placement [5].

There is no current research that compares the use of prophylactic Dobhoff with reactive Dobhoff. One study reports that those without a feeding tube can maintain nutrition similar to those with a feeding tube [6]. This study suggests a high rate of unnecessary PEG placement when done prophylactically in patients with HNC.

Risk factors

Some studies looked to identify patient characteristics, which correlate with the need for nutritional support during treatment. One study classified patients receiving bilateral neck treatment and/or CRT to be at the greatest risk of requiring modiﬁed diets and feeding tube use early post-treatment [7]. A systematic review found patient age as the only significant risk factor for requiring ETF. A correlation was seen between radiation dose to the inferior constrictors and long-term PEG dependence. Patients who maintain oral intake or perform swallowing exercises during treatment are more likely to have shorter PEG dependence [3]. Further research to compare patients receiving CRT vs. RT would be beneficial to further characterize high-risk groups.

PEG vs. Dobhoff

The majority of the research done on HNC and the use of feeding tubes looks specifically at the use of PEG tubes. One retrospective study suggested a PEG tube was required for longer periods of time and was associated with more persistent dysphagia and an increased need for pharyngoesophageal dilatation [8]. A randomized trial showed nutritional support with both PEG and Dobhoff was equal, and there were no significant differences in overall complication rates [9]. The cost of a PEG tube is 10 times that of a Dobhoff. The same study found the duration of use of PEG tubes to be significantly longer. They found no evidence to support the routine use of PEG tubes over Dobhoff.

The median weight loss decreased by 8.7% after the patients had a Dobhoff placed. (12.9% down to 4.2%). Additionally, at the six-month follow-up, all 11 PEG patients still had their feeding tube in place (23.4%) while no patients required a Dobhoff (n=0).

Lack of proper prospective research

There is a need for more recent prospective research looking at the optimal timing of Dobhoff placement during RT for HNC. There are few studies done within the past five years available to apply to current patient care. Studies like this can be used as guidance for in-clinic Dobhoff placement applications to other institutions. This data can help guide future clinical practice when taking care of patients during RT for HNC. This study is limited by its retrospective nature and by its lack of a comparator arm.

## Conclusions

This retrospective study found reactive Dobhoff placement to be a safe and effective strategy for nutritional support during RT for HNC. In-clinic placement of a Dobhoff can efficiently maintain nutrition support during treatment. The placement procedure is well-tolerated and there were no complications associated with having the Dobhoff during treatment. Correct placement by the APRN on the first attempt at placement was achieved in most patients. The median weight loss during treatment decreased after the patient had a Dobhoff placed.

Dobhoff feeding tubes can be used as a less invasive intervention for nutritional support with shorter long-term dependence. This study can be used as guidance in determining proper nutritional support during treatment for HNC.
